# Type and capacity of glucose transport influences succinate yield in two-stage cultivations

**DOI:** 10.1186/s12934-018-0980-1

**Published:** 2018-08-28

**Authors:** L. Kyselova, D. Kreitmayer, A. Kremling, K. Bettenbrock

**Affiliations:** 1 Team Experimental Systems Biology, Max Planck Institute for Dynamics of Complex Technical Systems, Sandtorstr.1, 39106 Magdeburg, Germany; 2 Systembiotechnologie, Technische Universität München, Bolzmannstr. 15, 85748 Garching, Germany

**Keywords:** Succinate production, Two stage cultivations, Glucose transport, Flux balance analysis, ATP, Pck

## Abstract

**Background:**

Glucose is the main carbon source of *E. coli* and a typical substrate in production processes. The main glucose uptake system is the glucose specific phosphotransferase system (Glc-PTS). The PTS couples glucose uptake with its phosphorylation. This is achieved by the concomitant conversion of phosphoenolpyruvate (PEP) to pyruvate. The Glc-PTS is hence unfavorable for the production of succinate as this product is derived from PEP.

**Results:**

We studied, in a systematic manner, the effect of knocking out the Glc-PTS and of replacing it with the glucose facilitator (Glf) of *Zymomonas mobilis* on succinate yield and productivity. For this study a set of strains derived from MG1655, carrying deletions of *ackA*-*pta, adhE* and *ldhA* that prevent the synthesis of competing fermentation products, were constructed and tested in two-stage cultivations. The data show that inactivation of the Glc-PTS achieved a considerable increase in succinate yield and productivity. On the other hand, aerobic growth of this strain on glucose was strongly decreased. Expression of the alternative glucose transporter, Glf, in this strain enhanced aerobic growth but productivity and yield under anaerobic conditions were slightly decreased. This decrease in succinate yield was accompanied by pyruvate production. Yield could be increased in both Glc-PTS mutants by overexpressing phosphoenolpyruvate carboxykinase (Pck). Productivity on the other hand, was decreased in the strain without alternative glucose transporter but strongly increased in the strain expressing Glf. The experiments were complemented by flux balance analysis in order to check the observed yields against the maximal theoretical yields. Furthermore, the phosphorylation state of EIIA^Glc^ was determined. The data indicate that the ratio of PEP to pyruvate is correlating with pyruvate excretion. This ratio is affected by the PTS reaction as well as by further reactions at the PEP/pyruvate node.

**Conclusions:**

The results show that for optimization of succinate yield and productivity it is not sufficient to knock out or introduce single reactions. Rather, balancing of the fluxes of central metabolism most important at the PEP/pyruvate node is important.

**Electronic supplementary material:**

The online version of this article (10.1186/s12934-018-0980-1) contains supplementary material, which is available to authorized users.

## Background

**Fig. 1:**
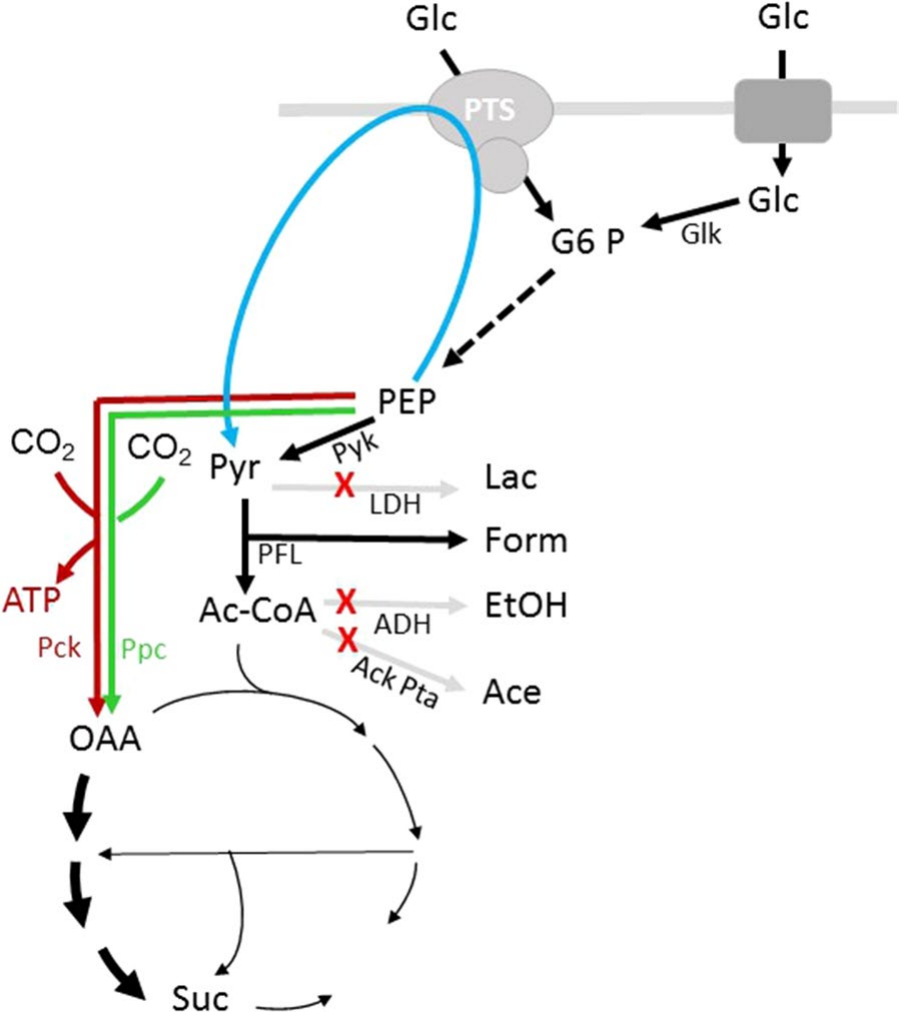
Scheme of anaerobic glucose metabolism in *E. coli*. Shown are reactions involved in anaerobic glucose metabolism. Only reactions important for the study at hand are shown. Deletions present in all mutant strains are marked by red crosses. Grey arrows indicate that the respective fermentation products are absent or strongly reduced in the mutant strains. The blue arrows marks the Glc-PTS that is present in some of of the strains. The red arrow indicates the reaction catlyzed by Pck, while the green arrow indicates the reaction of the Ppc. ATP and NAD are not show in the figure with exeption of the ATP gained by Pck

Biotechnological production of fine chemicals has gained increasing interest during the last years. Succinate has been ranked within the top 12 bio-based products by the US Department of Energy [1] and hence there are numerous efforts to produce succinate biotechnologically. Under anaerobic conditions, succinate is formed by *E. coli* as one of the products of mixed acid fermentation together with acetate, lactate, formate and ethanol [2]. Under aerobic conditions, succinate is an intermediate of the tricarboxylic acid cycle but it is not excreted. As succinate is produced only in minor amounts during natural fermentation, different strategies have been developed in order to improve the efficiency of succinate production by *E. coli*. Common to anaerobic strategies is prohibiting the production of the competing fermentation products acetate, formate and lactate by deletion of genes involved in their production (Fig. 1). This is often complemented by overexpression of endogenous or heterologous genes catalyzing oxaloacetate production from PEP or pyruvate to enhance metabolic fluxes towards succinate (see [3–6] and references therein). A major problem of all these strategies is that prohibiting the production of competing fermentation products strongly impairs growth under fermentative conditions. Hence, most engineered strains are incapable of fermentative growth in defined medium with glucose as sole carbon source. Many studies have hence been performed with complex media or have applied two stage strategies with an aerobic growth phase followed by an anaerobic production phase. Usage of complex medium however complicates the analysis of strain behavior as it is basically unknown which medium components are used and how they affect fluxes in the cell [7–9]. Also it complicates the calculation of yields.

Apart from anaerobic strategies of succinate production also aerobic and microaerobic strategies have been applied. In these strategies the production of acetate is prohibited, accompanied by a block in the tricarboxylic acid cycle and an enhancement of the glyoxylate shunt [10–13]. Aerobic strategies were motivated to overcome the severe growth problems of succinate production strains under anaerobic conditions. The mayor drawback of aerobic strategies is the low maximal theoretical yield of only 1 mol succinate per mol of glucose [11], which is significantly lower than for anaerobic conditions.

The Glc-PTS represents the major glucose uptake system in *E. coli*. It couples uptake with concomitant phosphorylation of glucose. The phosphodonor of the PTS is phosphoenolpyruvate (PEP) which is converted to pyruvate by this reaction (Fig. 1). The phosphoryl group from PEP is transferred by a phosphoryl transfer chain consisting of the PTS proteins EI, HPr, EIIA^Glc^ and EIICB^Glc^ until it ultimately is transferred to the incoming substrate glucose [14]. Mutations in *ptsG*, the gene encoding the EIICB^Glc^, were isolated as spontaneous mutations leading to higher succinate production rates. This mutation was also predicted to be favorable by theoretical methods [6, 11, 15, 16].

One mole of PEP is converted to pyruvate for each mole of internalized and phosphorylated glucose. PEP is an essential precursor of succinate and this is why inactivation of the PTS improves succinate production in *E. coli* [8, 17]. Strains lacking the Glc-PTS or the PTS in general are characterized by slow growth and low glucose uptake rates. Besides the Glc-PTS, glucose can be taken up by several other uptake systems, both PTS-systems (ManXYZ) [18] and non-PTS systems, the most important being MglABC, GalP and MalEFG [19–22]. Under natural conditions these systems can replace the PTS only marginally. Wang and coworkers [23] reported that overexpression of galactose permease (*galP*) in a PTS^−^ strain led to improved succinate production. Another strategy for improving cell growth and glucose uptake, and therefore also for increasing succinate production in PTS mutants, is the usage of the glucose facilitator protein, Glf of *Z. mobilis* [24–26], that allows an efficient uptake of glucose into cells by facilitated diffusion.

Although mutations of the Glc-PTS as well as introduction of alternative glucose transporters have been reported in a number of different studies, to our knowledge no systematic analysis of the effects of glucose uptake on succinate production have been reported.

In this article, we systematically compared a set of *E. coli* strains, distinguished by differences in glucose uptake, for the production of succinate in a two-stage strategy. Different knockout strains were analyzed for their growth capabilities, glucose consumption as well as the production of succinate and other by-products. A two-stage strategy was chosen to profit from the high theoretical yield under anaerobic conditions while at the same time circumvent the severe anaerobic growth defects of the test strains.

Common to all strains are knock outs of *ldhA*, *ackA*-*pta* and *adhE*. The strains differ with respect to glucose uptake: while one of the strains, KBM151101, still has the native Glc-PTS, this system (*ptsG*) was deleted in SB2. It is unknown which system is used for glucose uptake in SB2. To improve glucose uptake, the glucose facilitator, Glf, from *Z. mobilis* was introduced into SB2, giving rise to strain KBM1673. In addition, we overexpressed PEP carboxylase (Ppc) or PEP carboxykinase (Pck), important enzymes in the PEP to oxaloacetate conversion in the test strains.

To understand the observed behavior in more detail, the growth assays were complemented by determination of the intracellular ATP levels and by determination of the phosphorylation state of EIIA^Glc^. The experimental studies were completed by theoretical analyses of optimal growth and product formation of the strains using flux balance analysis (FBA). The data illustrate that the type of glucose uptake indeed is important for achieving a high succinate yield but besides this, it is also important to balance fluxes especially at the pyruvate/PEP node.

## Methods

### Bacterial strains and growth conditions

Strains used in this study were *E. coli* MG1655 and mutants thereof listed in Table 1. Mutant strains were constructed by homologous recombination [27] or by P1 transduction [28]. To integrate the glucose facilitator, Glf, of *Zymomonas mobilis* into the genome of *E. coli*, gene *glf*, encoding the glucose facilitator, was amplified together with *glk* from plasmid pF-71 (kind gift of G. Sprenger, University of Stuttgart). The constitutive *scrK* promoter was amplified from pCS26-scrK [29] and fragments were cloned into pAH162 [30] using the Gibson Assembly Cloning Kit from NEB. *glk* was deleted from the construct using the Q5 site directed mutagenesis kit from NEB. The corresponding plasmid pAH162-scrK_P_-glf was integrated into the chromosome of SB2 as described by Haldimann and Wanner [30]. For construction of pPckA the coding sequence of *pckA* was amplified from chromosomal DNA of MG1655 and cloned into the expression vector pRR48c [31]. Primers used for plasmid and strain constructions are indicated in Additional file 1: Table S1.

**Table 1:** Strains and plasmids used in this study

Strain	Relevant genotype	Source or reference
MG1655	Wildtype	FRB426 (Blattner lab)
KBM151101	MG1655 Δ*ackA*-*pta* Δ*adhE* Δ*ldhA* Δ*lacI*-*A*	This study
SB2	MG1655 *ΔackA*-*pta ΔadhE ΔldhA ΔlacI*-*A ΔptsG*	This study
KBM1673	MG1655 *ΔackA*-*pta ΔadhE ΔldhA ΔlacI*-*A ΔptsG* attphi80::pAH162_*scrKp*_*glf*_*Z*.mobilis_	This study
Plasmid		
pPckA	*pckA* from *E. coli* MG1655 in pRR48	This study
pEcPpc	*ppc* from *E. coli* in pTrc99A	[32]

*E. coli* cells from agar plates were grown in LB_0_ medium (10 g/l tryptone, 5 g/l yeast extract, 5 g/l NaCl) for 5–6 h at 37 °C. Afterwards the culture was diluted 1:100 or 1:50 into minimal salts medium [33] with 4 g/l glucose and incubated aerobically on a rotatory shaker (250 rpm) at 37 °C for 8–12 h. The culture in mid exponential growth phase was centrifuged and the washed cells were inoculated to 10^9^ cells/ml into 50 ml fresh minimal medium containing 4 g/l glucose and 2 g/l NaHCO_3_. For anaerobic cultivations the culture was split into aliquots and cells were incubated in tightly sealed 5 ml glass vials at 37 °C without agitation.

### Analytical techniques

Optical density (OD_420_) was determined spectrophotometrically at 420 nm (Ultrospec3000, Amersham, Bioscience). An OD_420_ of 1 corresponds to 5*10^8^ cells/ml. To assure a linear correlation of OD_420_ and biomass, cell cultures were diluted before the measurement to obtain a measurement value between OD 0.05 and 0.5. The culture samples were harvested by quick centrifugation (2 min) and supernatants were used for further analysis. For organic acids measurements such as succinate, pyruvate, formate, acetate and orotate the supernatant was filtered and analyzed by HPLC using an Agilent 1100 Series system equipped with DAD detector (Agilent Technologies) and an Inertsil ODS-3 column (Gil Science Inc.). As mobile phase 0.1M NH_4_H_2_PO_4_, pH 2.6 solution was used at a flow rate of 1 ml/min. Concentrations in the samples were calculated based on standard solutions run under identical conditions.

The amounts of glucose, ethanol and acetate were determined using the respective enzymatic test kits d-glucose-HK, ethanol and acetc acid of Megazyme International Ireland.

Glucose uptake was determined by linear regression analysis by the method of least squares based on glucose levels determined in the interval between 0 and 48 h. Yield and productivity were determined independently. Yield was determined by plotting the time series of glucose concentrations in the medium against the concentrations of succinate at the given time points. Yield was estimated from the line of best fist applying total least square regression analysis to this biomass-glucose diagram. Uptake rate for glucose and production rate for succinate were determined analogously from the time course data.

### Determination of EIIA^Glc^ phosphorylation

The EIIA^Glc^ phosphorylation state was determined by SDS-PAGE and Western Blotting as described [29, 34]. Samples were taken from mid-exponential phase under aerobic conditions and after 4 h of cultivation under anaerobic conditions, respectively.

### Measurement of ATP concentration in cells

100 µl of growing culture was immediately quenched in 900 µl of boiling water and the mixture was incubated at 99 °C for 10 min. Afterwards the sample was centrifuged at 13,000 rpm for 10 min and the ATP concentration of the supernatant was determined using the ATPLite kit (PerkinElmer) according to the instructions of the manufacturer. The concentration was normalized by taking into account the cell density in the respective sample.

### Gene expression analysis by RT-qPCR

About 1.5*10^9^ cells from exponential aerobic growth phase or after 2–4 h incubation in the anaerobic phase were quenched in twice the volume of RNA protect Bacterial Reagent (Qiagen), vortexed for 5 s and incubated at room temperature for 5 min. Cells were pelleted by centrifugation, the supernatant was discarded and the pellet was stored at − 80 °C. RNA was prepared using the Master Pure RNA Purification Kit (Epicentre). RNA concentration and purity was determined using the NanoDrop spectrophotometer (Thermo Scientific).

mRNA was transcribed into cDNA by using the RevertAid H Minus First Strand cDNA synthesis Kit (Thermo Fisher Scientific). Quantitative PCR of different cDNA samples was performed using the MesaGreen qPCR Master Mix Plus (Eurogenetec) with SYBR Green as detection agent and the Rotor-Gene 6000 (Corbett Life Science). Sequences of the primers used are listed in Additional file 1: Table S1. Amplification conditions were: 95 °C for 10 min, 40 cycles at 95 °C for 15 s and 60 °C for 1 min. A negative control without template was conducted for each primer pair in each PCR run and a control for DNA contamination was performed for each RNA sample used. Quantification was performed by relative quantification to housekeeping genes (*rpoD*, *yhbc* and *ihfB*) applying the ΔΔCt method [39, 40] with efficiency correction.

### Flux balance analysis

Flux Balance Analysis (FBA) was carried out using the CobraToolbox v2.0 [35] and an adapted version of the included ecoli_core_model [36].

The exact model structure is given in the Additional file 2. The Cobra Toolbox was run in MATLAB 2014b using Gurobi optimizer version 6.5.0.

### Simulation of EIIA^Glc^ phosphorylation

To analyze the measured phosphorylation levels of EIIA^Glc^ we used a mathematical model that considers substrate uptake for PTS and for non-PTS carbohydrates [37]. The model was developed for aerobic growth. It describes glycolysis with a reduced number of components and reactions. It comprises five components: glucose-6-phosphate, fructose-6-phosphate, PEP, pyruvate and EIIA^Glc^. The components are linked by five reactions: substrate uptake, representative reaction of the upper part of glycolysis, pyruvate kinase, pyruvate dehydrogenase, and by-product excretion. In case of PTS sugars, the uptake reaction requires 1 mol of PEP and produces 1 mol of pyruvate. The model was validated with experimental data for different growth rates and shows satisfactory agreement between simulation and experimental data [37]. The main outputs of the simulations are relationships between the steady-state values of the components as a function of the growth rate μ. In this way, a characteristic curve for the degree of phosphorylation of EIIA^Glc^ in dependence on the growth rate is obtained.

The model was adapted to the various mutant strains used in this study and to the anaerobic case. In case the PTS is missing, the uptake reaction was decoupled from PEP to pyruvate conversion. To mimic the mutations, the drain of by-products e.g. acetate or lactate was omitted. In this way, the modifications focus on the pyruvate node and its drain in biosynthesis. To validate the model for the anaerobic case, the uptake rate is taken as input into the model and only the kinetic parameter for the pyruvate drain reaction was fitted to get a good agreement of the measured degree of phosphorylation of EIIA^Glc^ with the simulation results. In the anaerobic case, only one growth rate was analyzed for each mutant. Therefore, each curve generated in the simulation could be verified with only one data point. Since under anaerobic conditions the flux through glycolysis is higher than during aerobic growth, the maximal uptake rate for strains with a PTS was increased by 30%. The simulation also allows us to determine the PEP/pyruvate ratio. The analysis of the characteristic curves for the PEP/pyruvate ratio is the basis for a correlation with the pyruvate produced by the strains during the experiment.

## Results

### Characterization of aerobic growth in isogenic mutant strains with different glucose uptake systems

As described in the introduction, there are numerous studies dealing with improving succinate production in *E. coli*. While these studies describe improvements of succinate production by knock-out or overexpression of multiple genes, a systematic investigation of the effects of a certain knock-out is lacking. *ptsG* or *ptsI* were knocked out in several strategies [8, 11, 17, 38–42]. These mutations dramatically slow down growth with glucose as carbon source and hence diminish productivity. We investigated, in a systematic way, the effects of knocking out *ptsG* and of introducing an alternative glucose transporter on succinate production. The strain analyzed, KBM151101, has deletions in *adhE*, *ldhA* and *ackA*-*pta* (Fig. 1). As KBM151101 is not able to grow anaerobically in mineral salts medium with glucose as sole carbon source, a two-stage cultivation strategy was employed. Strain SB2 was constructed by knocking out *ptsG* in KBM151101. In strain KBM1673 we replaced the Glc-PTS with the PTS-independent glucose facilitator, Glf, of *Z. mobilis*. This transporter enables uptake of glucose by facilitated diffusion and is hence independent of ATP, PEP or proton gradient. Glucose uptake via Glf should not affect energy charge.

We first analyzed growth of the wild-type strain and the mutants under aerobic batch conditions, in minimal medium with glucose (Table 2). The mutant KBM151101 showed a similar growth behavior as the wild-type. As the deletions in KBM151101 affect the anaerobic fermentative pathways, this is in agreement with current knowledge. As expected, SB2, the *ΔptsG* strain incapable of uptaking glucose by the glucose-PTS, grew significantly slower on glucose (µ = 0.26 h^−1^, compared to µ = 0.72 h^−1^ for MG1655). In KBM1673 the introduction of Glf from *Z. mobilis* complemented the *ptsG* deletion, although not completely. Glf is hence able to largely replace PtsG in glucose uptake.

**Table 2:** Growth rates of wild-type and mutant strains under aerobic conditions

Strain	Growth rate [1/h]
MG1655	0.72 ± 0.01
KBM151101	0.73 ± 0.03
SB2	0.26 ± 0.03
KBM1673	0.50 ± 0.07

Data are derived from two independent experiments

### Characterization of anaerobic growth and succinate production

**Fig. 2:**
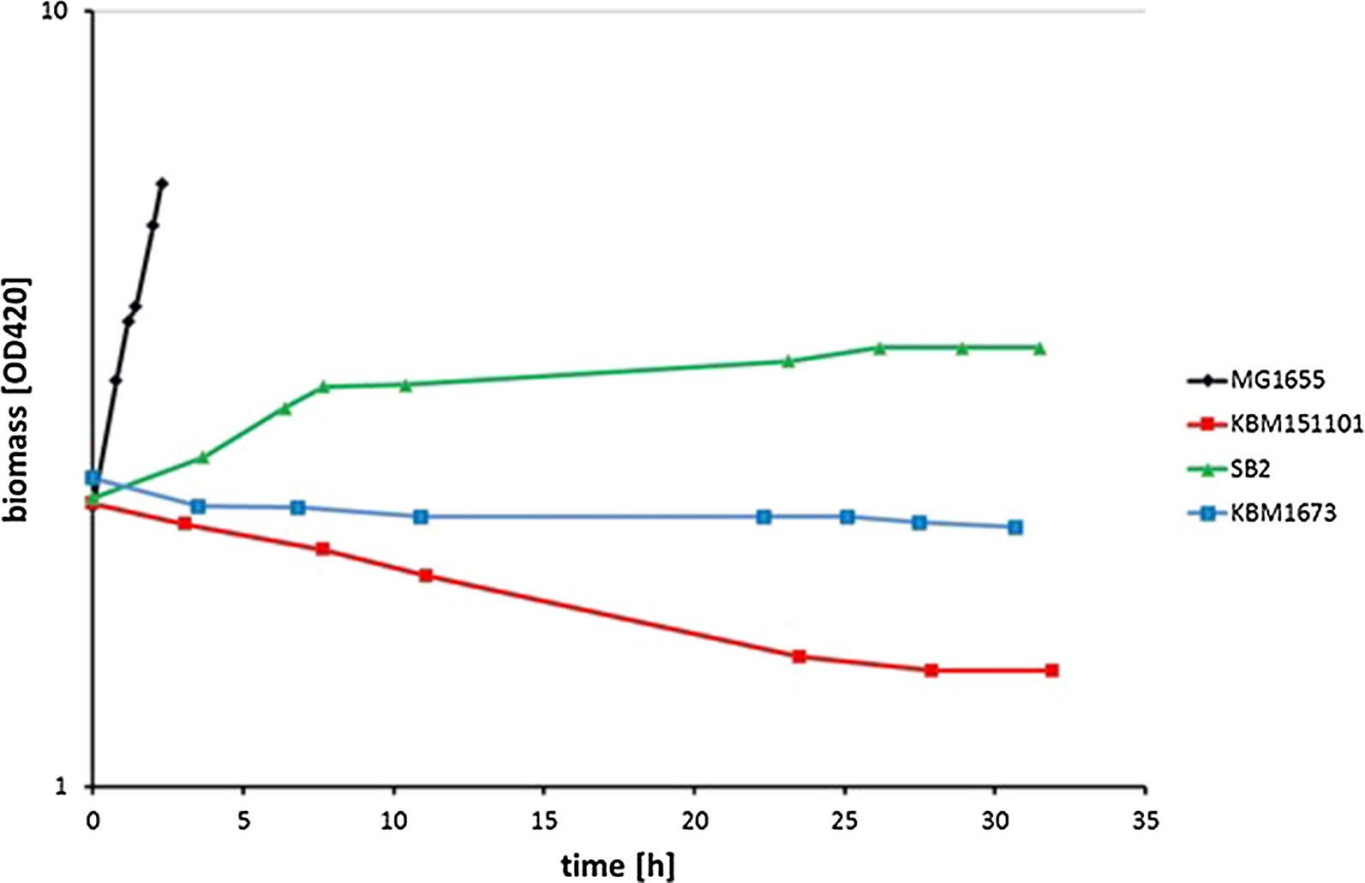
Course of biomass concentrations during the anaerobic phase of two-stage cultivation. Shown are biomass measurements for the wild tpye MG1655 (black), KBM151101 (red), SB2 (green) and KBM1673 (blue). Biomass concentration is plotted on a logarythmically scaled axis. At the start of the anaerobic phase, all strains were inoculated to the same cell density (OD_420_ ~ 2). While MG1655 shows quick anaerobic growth, growth of all mutant strains is strongly impaired. The data shown are derived from one exemplary experiment. At least three repeats have been carried out for all strains amd average data concerning growth rates are given in Table 3

Two-stage cultivations are useful if growth during the production phase is impaired or slow. During the first phase, the cells are maintained in an environment favorable for fast growth and for biomass accumulation, while no or only low amounts of product are produced. This phase is then followed by a second, production phase. In this case, growth of the mutant strains under fermentative conditions is impaired. Hence, an aerobic growth phase was introduced before the culture was shifted to an anaerobic succinate production phase. Cells from mid-exponential aerobic growth were harvested by centrifugation and incubated anaerobically in fresh minimal medium with 4 g/l glucose and 2 g/l carbonate. The anaerobic growth behavior of *E. coli* MG1655 and the mutants is presented in Fig. 2. While MG1655 continued to grow in the second, anaerobic stage (μ = 0.4 h^−1^), the cell number of KBM151101 decreased slowly but clearly (Fig. 2). In contrast, the *ptsG* mutant, SB2, grew after the shift, albeit extremely slow (µ = 0.02 h^−1^). Growth stopped after about 10 h in the anaerobic phase, but the cell number was not decreasing until the end of the experiment. Notably, the Glf^+^ derivative, KBM1673, showed an intermediated behavior. Cell numbers of these strains stayed almost constant during the anaerobic phase.

Glucose consumption rates and by-product yields for the second phase of two-stage cultivations are presented in Table 3. The anaerobic glucose consumption rates of all mutant strains were significantly lower than that of MG1655. While KBM151101 consumed only small amounts of glucose, SB2 carrying the *ptsG* deletion showed improved anaerobic glucose utilization. This was against expectation and hints to differences in the expression of glucose uptake systems or alternative metabolic pathways in *ptsG* mutants compared to KBM151101 under anaerobic conditions. SB2 synthesized succinate as a major anaerobic fermentation product with a high yield of 1.3 mol/mol glucose. The anaerobic glucose uptake rate of KBM1673, expressing Glf, was higher than that of KBM151101, but unexpectedly did not reach the uptake rate of SB2. Also, succinate production rates were intermediary for this strain. The data imply that in the anaerobic phase of two stage cultivations glucose uptake is possible by expression of (a) so far unknown uptake system(s) and that the presence of uptake systems which are efficient under aerobic conditions (like Glf) does not necessarily improve anaerobic glucose uptake in strains unable to grow.

**Table 3:** Strain performance in the second, anaerobic phase of two-stage cultivations

Strain	µ [1/h]	Glc_Up [mM/(g*h)]	Y_Suc [mol/mol_Glc_]	Y_For [mol/mol_Glc_]	Y_Pyr [mol/mol_Glc_]	Prod_Suc [mM/(g*h)]
MG1655	0.37 ± 0.011	9.07 ± 0.94	0.29 ± 0.02	0.28 ± 0.03	nd	2.62 ± 0.18
KBM151101	− 0.013 ± 0.002	0.15 ± 0.03	0.10 ± 0.05	0.12 ± 0.16	0.07 ± 0.05	0.02 ± 0.01
SB2	0.011 ± 0.001	1.10 ± 0.19	1.24 ± 0.01	0.38 ± 0.02	nd	1.29 ± 0.25
SB2/pPckA	0.004 ± 0.001	0.59±0.13	1.42 ± 0.03	0.38 ± 0.01	nd	0.83 ± 0.18
SB2/pEcPpc	− 0.012 ± 0.002	0.23 ± 0.07	1.08 ± 0.08	0.11 ± 0.03	0.05 ± 0.01	0.25 ± 0.08
KBM1673	− 0.003 ± 0.000	0.64 ± 0.10	0.83 ± 0.04	0.47 ± 0.01	0.22 ± 0.02	0.53 ± 0.08
KBM1673/pPckA	0.068 ± 0.002	2.68 ± 0.12	0.92 ± 0.01	0.25 ± 0.01	0.35 ± 0.02	2.76 ± 0.10
KBM1673/pEcPpc	− 0.007 ± 0.001	0.18 ± 0.05	1.05 ± 0.10	0.37 ± 0.03	0.06 ± 0.02	0.19 ± 0.05

All data refer to the second, anaerobic phase of two-stage cultivations. µ denotes the growth rate in doublings per hour. Negative values for µ result from decreasing OD_420_ over time which is most probably related to cell death. Glc_Up denotes the specific glucose uptake rate and Prod_Suc denotes the succinate productivity. g means g dry cell weight. The yields Y_Suc, Y_For, Y_Pyr stand for the moles succinate, formate and pyruvate respectively, produced per mole of glucose consumed. Errors indicated refer to SD. Reduction of biomass in some strains results in a negative growth rate. Glucose uptake and succinate productivity were calculated by weighted linear least squares. All results are the average of at least three independent biological repeats with exception of MG1655 with only two replicates. nd means that the product could not be detected

Notably, succinate yield is inversely linked to pyruvate production (Table 3). KBM1673 produced significant amounts of pyruvate while no pyruvate production was detected for SB2. Pyruvate production is hence not only influenced by PTS uptake of glucose but is also related to the uptake rate by non-PTS systems and to the fluxes in central metabolism.

### Overexpression of PEP carboxykinase and PEP carboxylase

PEP carboxykinase (Pck) and PEP carboxylase (Ppc) are important gluconeogenic or anaplerotic enzymes, respectively. Both enzymes are able to catalyze the carboxylation of PEP to oxaloacetate. Compared to Ppc, Pck requires higher concentrations of CO_2_ to catalyze the reaction of PEP to oxaloacetate [43–45]. Oxaloacetate production by Pck is accompanied by the gain of one molecule of ATP. Previous publications showed an increase in succinate production resulting from overexpression of the native *E. coli ppc* gene [32, 46]. Introduction of the gene encoding Pck of *A. succinogenes* into *E. coli* or overexpression of the native *E. coli pck* gene improved succinate production [43, 47], too. Overexpression of Pck provides the cells with additional ATP and leads to better growth [44].

We hence tested the effect of overexpressing the native Pck and Ppc of *E. coli* in our test strains (Table 3). Introduction of Pck into SB2 and KBM1673 increased succinate yield by about 10%. Notably, Pck overexpression strongly increased productivity in KBM1673. In SB2, on the contrary, a slight drop in productivity was observed. Ppc overexpression had a low positive effect on succinate yield in KBM1673 but not in SB2 and productivity decreased in both strains with introduction of Ppc. Obviously, enhancement of the PEP to oxaloacetate reaction is positive for succinate productivity but only if catalyzed by Pck. This might be reasoned in the additional ATP generated by the Pck reaction.

### Determination of ATP level and *pck* expression

**Fig. 3:**
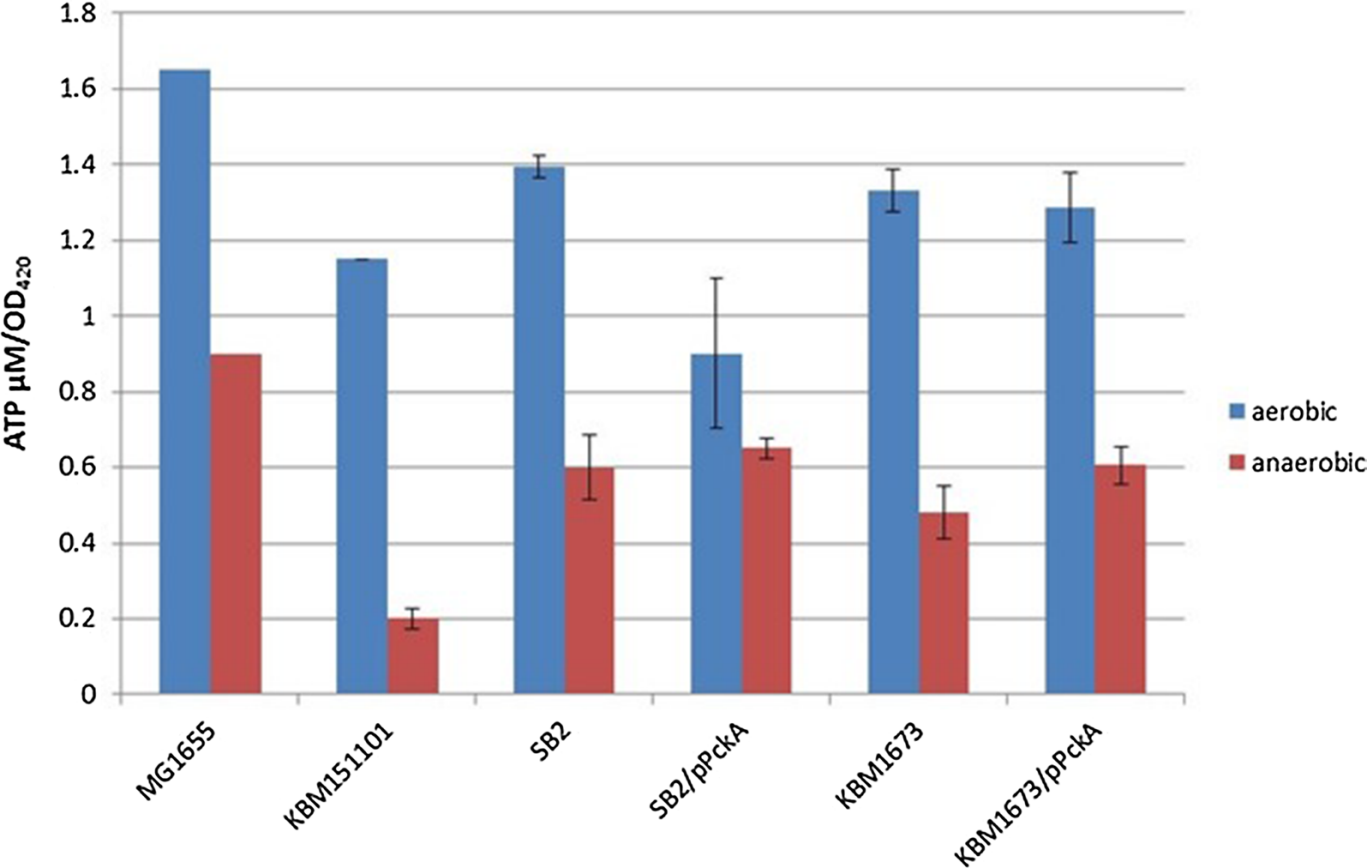
Measurement of ATP level of strains MG1655 and mutants. Shown are the concentrations of intracellular ATP in the different strains during the aerobic (blue) and anaerobic (red) phase of two-stage cultivations. Samples were taken during the exponential phase of aerobic growth and after 4 h in the anaerobic phase. Data are derived from two independent experiments with exception of MG1655 with only one repeat

ATP is an important factor determining growth and also succinate production. We measured the ATP levels of the mutant strains in the aerobic and anaerobic phase of two-stage cultivations. The aerobic ATP level was approximately the same in all strains (Fig. 3) with the only exception of SB2/pPck that shows a slightly lower ATP concentration. Based on ANOVA analysis this difference is not significant, though (Additional file 1: Figure S2) Under anaerobic conditions, the ATP concentration was highest in MG1655 reflecting its good growth under these conditions. The ATP levels of the mutant strains were lower than that of MG1655. The lowest concentration was observed for KBM151101. Based on ANOVA this difference can be regarded as significant (Additional file 1: Figure S2). This low ATP concentration could be the reason for growth impairment of this strain and also for the observed cell death during the second, anaerobic phase. SB2 and KBM1673 showed slightly lower ATP levels than MG1655 but significantly higher levels than KBM151101. It is tempting to speculate, that in these strains, especially in SB2, PckA is expressed in the anaerobic phase and allows for some flux from PEP to succinate coupled to ATP synthesis. For both strains overexpression of PckA results in slightly higher ATP levels probably reflecting increased PEP to oxaloacetate fluxes.

**Fig. 4:**
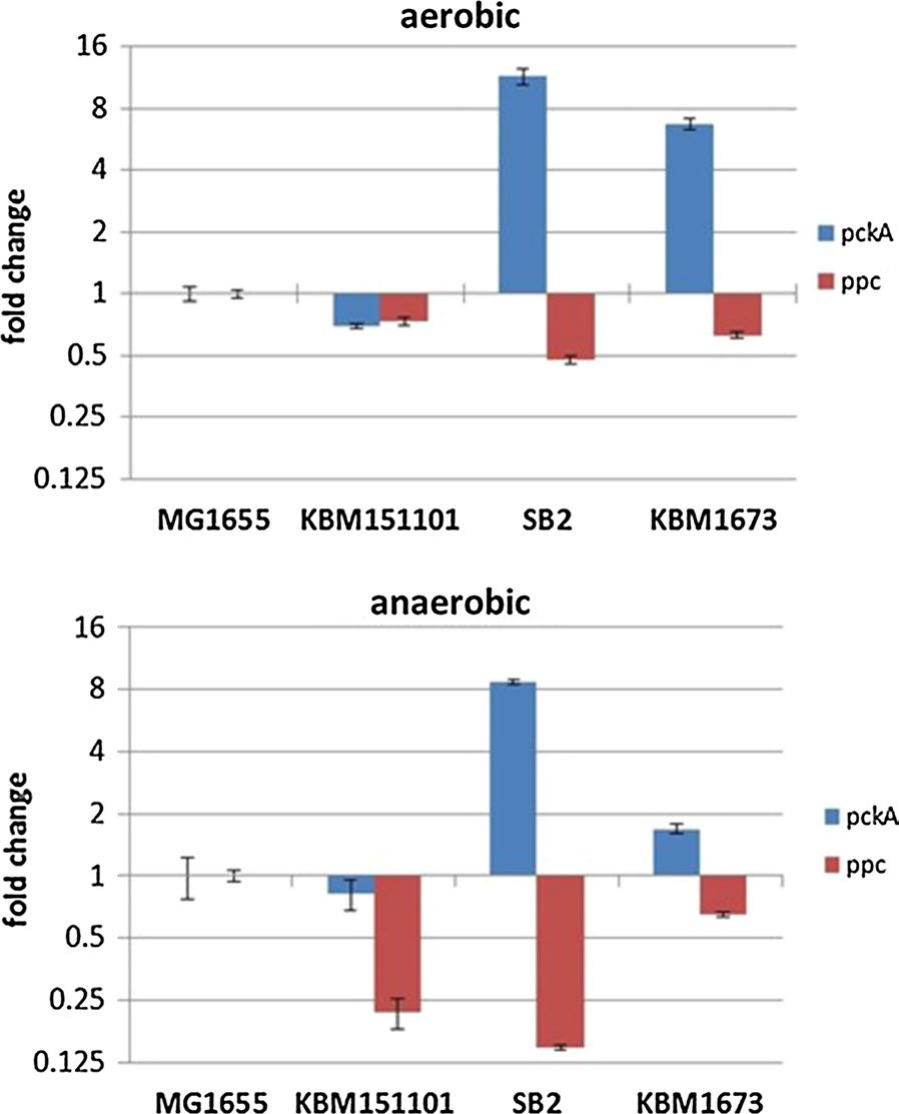
Analysis of *pckA* and *ppc* expression by Real Time RT PCR. Shown are the relative expression levels of *pckA* (blue) and *ppc* (red) determined for the different strains under aerobic and anaerobic conditions. Samples were taken from the exponential growth phase of the aerobic stage and after 4 h (mutant strains) or 2 h (MG1655 in the anaerobic stage. Gene expression was analysed by the ∆∆Ct method with normalization to three housekeeping genes and to MG1655 under the respective condition. Y-axes are plotted logarithmically to be able to show also downregulation

To test the hypothesis of *pckA* being expressed in SB2, we performed Real Time RT PCR and determined *pckA* as well as *ppc* expression in the different mutants under aerobic as well as anaerobic conditions. As can be seen from Fig. 4, *pckA* is clearly upregulated in SB2 under aerobic as well as anaerobic conditions. Also for KBM1673 an upregulation of *pckA* is visible but only under aerobic conditions. *ppc* on the contrary is downregulated in KBM151101 and SB2 under anaerobic conditions.

### Evaluation of theoretical yields by flux balance analysis and conclusions for strain design

To understand the stoichiometric limits of succinate production, flux balance analysis was performed to determine theoretical yields (see Additional file 2). To calculate the maximal theoretical yield, only the pathways from substrates to the products were considered, but not growth. The general assumptions used here were anaerobic cultivation in minimal medium with glucose and carbonate. Glyoxylate shunt, pyruvate dehydrogenase complex (Pdh) and phosphoenolpyruvate synthase (PpsA) were considered to be inactive. This was reasoned in the assumptions that Pdh is not active under anaerobic conditions. PDH is inhibited by NADH and under anaerobic conditions, PFL is responsible for the cleavage of pyruvate. PpsA is a gluconeogenic enzymes and is repressed in the presence of glucose to avoid futile cycling [48, 49]. The glyoxylate shunt is downregulated during batch growth with glucose as carbon source [50–52]. For the simulation of the mutant strains, the respective gene knock-outs were considered. With carbonate as second substrate and without considering growth, a maximal yield of 1.7 $\frac{mol\, succinate}{mol \,glucose}$ is possible. However, to reach this value, glucose uptake is distributed on the PTS as well as on alternative (non-PTS) uptake systems with 71% uptake by the non-PTS system.

The importance of carbonate supplementation becomes obvious when comparing the theoretical optimum with and without carbonate addition. Without external CO_2_ supplementation, a yield of 1.3 $\frac{mol \,succinate}{mol \,glucose}$ is possible, if optimal balance between carbonate formation by glycolysis, pentose phosphate pathway, formate cleavage, and carbonate fixation during anaplerosis is achieved. For this, at least 33% of glucose uptake need to be catalyzed by non-PTS uptake systems. However, the main glucose uptake system of *E. coli* is the PTS which requires that at least 50% of glucose derived PEP is converted to pyruvate for glucose uptake. This constraint reduces the maximum yield to 1 $\frac{mol\, succinate}{mol \,glucose}$ independent of carbonate supplementation. Therefore, simulation studies indicate non-PTS uptake systems as highly favorable for succinate production. This prediction agrees well with the tremendous increase in product yield observed for SB2 compared to KBM151101.

In a second scenario, a slow growth of biomass was considered. Demanding a minimal biomass yield of 0.01 $\frac{gDW}{mmol \,glucose}$ to achieve a viable cell population, the maximal theoretical succinate yield is reduced to 1.4 $\frac{mol \,succinate}{mol \,glucose}$. This theoretical value is energy limited and requires acetate formation to supply sufficient ATP. One way to alleviate energy limitation is recruiting Pck for anaplerosis. Working in the reversed direction from PEP to oxaloacetate, Pck produces ATP. In contrast to Pck, the usual anaplerotic enzyme Ppc produces only inorganic phosphate and no ATP. Overexpression of Pck allows for a higher theoretical yield of 1.6 $\frac{mol\, succinate}{mol \,glucose}$, while still maintaining a minimal biomass yield of 0.01 $\frac{gDW}{mmol \,glucose}$. Under regular growth conditions Pck is regarded as a gluconeogenic enzyme whose expression is repressed in the presence of glucose [53, 54]. The carboxylating activity of Pck can occur in vivo, if the gene is overexpressed and if the carbonate concentration is high enough to shift the equilibrium towards oxaloacetate production [43, 55]. Our experiments showed a positive effect of Pck overexpression on succinate yield for SB2 and KBM1673. However, only for KBM1673 a positive effect on growth was observed.

In a third scenario, we considered a more natural objective function, namely the maximization of biomass. When considering the experimental validated strains, the best theoretical product yield is predicted for *ptsG* knockout plus *pckA* overexpression, predicting a product yield of 1.32 $\frac{mol \,succinate}{mol\, glucose}$ and a biomass yield of 0.028 $\frac{gDW}{mmol \,glucose}$. This theoretical biomass yield gives an upper boundary of growth, assuming that no limitations by maintenance metabolism, futile cycles and stress responses are present. However, while the corresponding strain KBM1673/pPckA almost reaches optimal biomass yield, there are other limitations on the flux: these limitations lead to the formation of pyruvate as a second fermentation product and thus decrease succinate yield. From FBA the reason for the pyruvate production by KBM1673/pPckA, that becomes obvious from the measurements, is not clear. One might speculate that the flux that can be channeled by Pck is too low to cope with the amount of PEP produced by glycolysis. The amount of PEP that can not be processed by Pck is converted to pyruvate and some fraction of this pyruvate is found in the outside medium.

### Analysis of mutant behavior by metabolic flux analysis

The results above are valid for optimal behavior, and describe the stoichiometric possibilities of the strains. Next, we considered the experimentally obtained uptake rates as input into the model. For these studies, maximization of growth or the biomass function was taken as objective function. In all cases, except SB2, the simulated growth rate was slightly higher than the observed growth rate. For SB2, growth could only be explained if allowing for Pck activity. This finding was also supported by *pckA* expression analysis presented in Fig. 4 and by mRNA Seq analysis (data not shown). The simulated growth rates are slightly higher since FBA gives an upper limit growth, as mentioned previously. Also, in some cases the simulation predicts secretion of additional by-products like glutamate, which were not measured in our experiments.

To evaluate how efficiently the carbon source is converted into product by each strain, we calculated individual theoretical yields considering the observed growth rate and the respective genetic background (see Table 4). Comparing our strains to this adapted value shows, that especially SB2 and SB2/pPckA are close to optimal product formation.

**Table 4:** Comparison of predicted and observed succinate yields for the mutant strains

	Observed Yxs [$\frac{gDW}{mmol\, glucose}$]	Observed Yps [$\frac{mol \,succinate}{mol \,glucose}$]	Strain adapted theoretical yield [$\frac{mol \,succinate}{mol\, glucose}$]	Percentage of adapted optimum [%]
KBM151101	0	0.10	1	10
SB2	0.010	1.24	1.59	78
SB2/pPckA	0.006	1.42	1.64	86
SB2/pPpc	0	1.08	1.71	63
KBM1673	0	0.83	1.71	48
KBM1673/pPckA	0.025	0.92	1.4	65
KBM1673/pPpc	0	1.05	1.71	61

Yxs: observed biomass yield (as shown in Table 3); Yps: observed succinate yield (as in Table 3); adapted theoretical yield given by the maximal yield predicted by FBA when constrained by observed biomass yield and genetic background; percentage of adapted optimum calculated by dividing the observed succinate yield by the strain adapted optimal yield

**Fig. 5:**
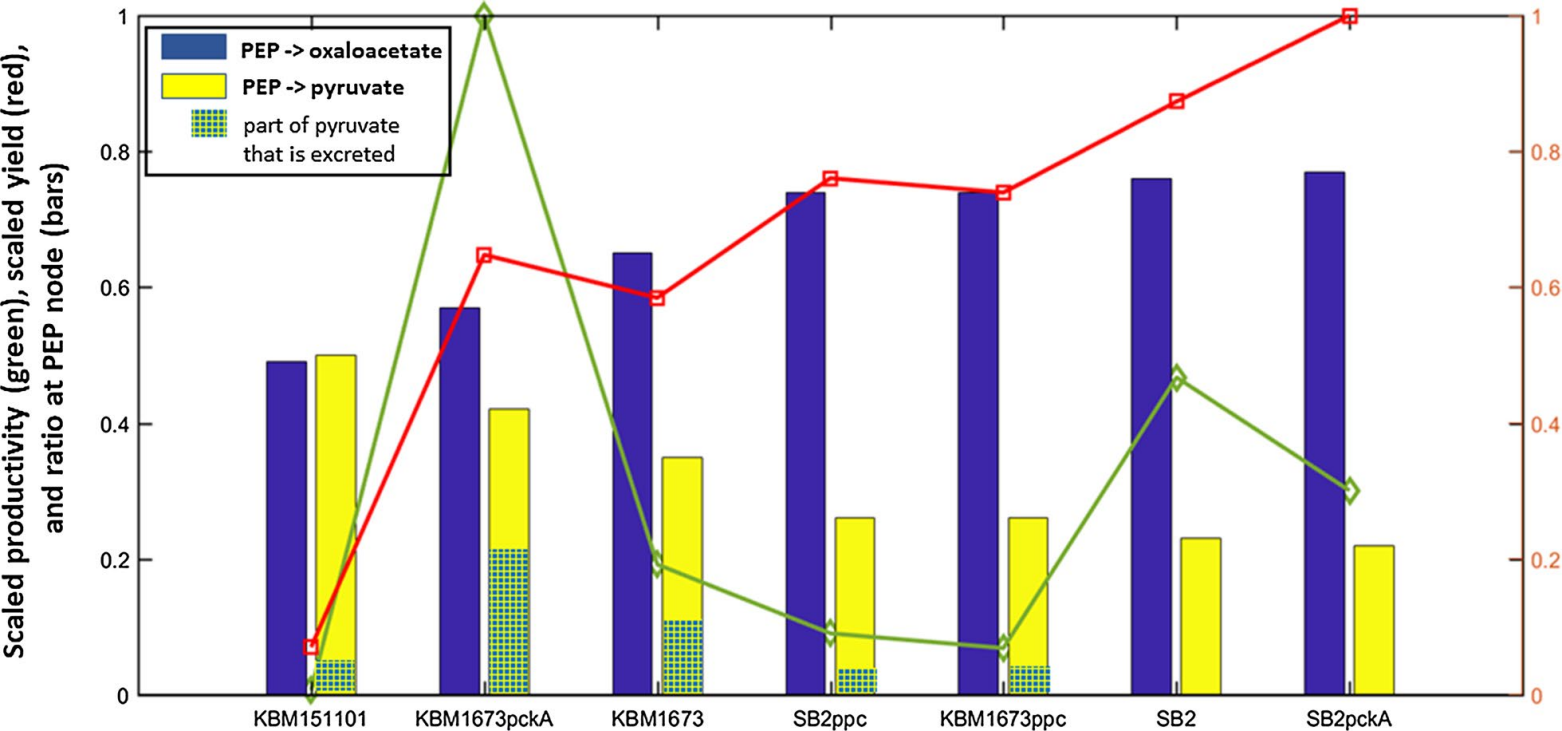
Flux ratio at node PEP. Bars show the relative flux from PEP to oxaloacetate (blue bars) and pyruvate (yellow bars) as determined by FBA; the hatched green part in the yellow bars indicate the fraction of the flux to pyruvate that is excreted into the medium as given by the measurement of extracellular pyruvate. Superimposed are shown the succinate yield (relative to the maximal yield obtained in this study) (red curve) and productivity (relative to the maximal productivity obtained in this study) (green curve)

Metabolic flux analysis was also used to analyze in more detail the fluxes at the PEP and pyruvate node. For this analysis glucose uptake rates as well as the production rates of extracellular succinate and pyruvate were taken into account. As can be seen from Fig. 5 for strains giving good succinate yields, low fluxes from PEP to pyruvate and high fluxes from PEP to oxaloacetate are predicted. No clear correlation between the fluxes at the PEP-pyruvate-oxaloacetate node and productivity is visible. Apart from KBM151101, a higher flux to pyruvate seems to be correlated with pyruvate excretion. Most probably, Pck or Ppc activity are important for the fluxes at the PEP-pyruvate node. The amount of PEP that can not be converted to oxaloacetate by these enyzmes will by converted to pyruvate and partly excreted. Data for KBM151101 are to be treated with care. As this strain takes up only very small amounts of glucose, measurements of glucose uptake and also of products formed are faulty, due to the detection limit of the assays applied.

### Determination of EIIA^Glc^ phosphorylation

EIIA^Glc^ is a central regulator of carbon metabolism. The unphosphorylated EIIA^Glc^ is an inhibitor for uptake of sugars such as maltose, lactose, and glycerol, an effect called catabolite repression [14]. Uptake of PTS substrates but also fast growth with non-PTS substrates results in a low EIIA^Glc^ phosphorylation state [29, 56], because the PEP to pyruvate ratio is low during fast growth. We analyzed the EIIA^Glc^ phosphorylation state in the different mutants in both stages of two-stage cultivations (Table 5). When grown on glucose, 95% of EIIA^Glc^ in an *E. coli* wild-type strain is unphosphorylated [29]. This was also observed for KBM151101 during aerobic growth. SB2, the *ptsG* mutant, showed increased EIIA^Glc^ phosphorylation under aerobic conditions, reflecting its slow growth and the lack of the Glc-PTS. KBM1673 shows an intermediary EIIA^Glc^ phosphorylation level, reflecting its intermediary growth rate and maybe also the fact that glucose uptake in this strain is catalyzed by the PTS-independent Glf.

**Table 5:** Phosphorylation state of EIIA^Glc^ during two-stage cultivations

Strain	EIIA^Glc^-P [%]	EIIA^Glc^-P [%]
	Aerobic stage	Anaerobic stage
MG1655	4.2 ± 1.3	4.9 ± 2.1
KBM151101	2.1 ± 0.8	4.5 ± 2.3
SB2	91.3 ± 1.3	80.3 ± 3.1
KBM1673	72.1 ± 12.5	23.3 ± 6.0
SB2/pPckA	83.4 ± 6.0	71.5 ± 3.5
KBM1673/pPckA	61.4 ± 12.3	56.9 ± 15.3

Shown are the EIIA^Glc^ phosphorylation levels in the different strains during the aerobic and anaerobic phase of two-stage cultivations. Samples were taken during the exponential phase of aerobic growth and after 4 h in the anaerobic phase. Data are derived from at least two independent experiments. Data from single growth curves as well as exemplary pictures of Western Blots are shown in Additional file 1: Table S2 and Figure S1

While the EIIA^Glc^ phosphorylation state is in good agreement with the growth rates for aerobic growth, it is more difficult to interpret the data from the anaerobic phase (Table 5). The wild type MG1655 showed a low EIIA^Glc^ phosphorylation level also under anaerobic conditions. Unexpectedly, a low EIIA^Glc^ phosphorylation level was also observed for KBM151101, although this strain did not grow during the anaerobic phase. Slow growth is normally coupled to high EIIA^Glc^ phosphorylation but not in this case. It has been hypothesized, that in addition to the PTS activity the EIIA^Glc^ phosphorylation level also reflects the PEP to pyruvate ratio in the cell [29, 57]. Given the fact that KBM151101 excreted pyruvate, a high concentration of pyruvate is expected within the cell, and might explain the low EIIA^Glc^ phosphorylation state. Intracellular pyruvate accumulation might also be responsible for the low EIIA^Glc^ phosphorylation in KBM1673. In SB2, the EIIA^Glc^ phosphorylation state was slightly lower under anaerobic than under aerobic conditions but still quite high. Again, this fits to the lack of pyruvate excretion by SB2.

**Fig. 6:**
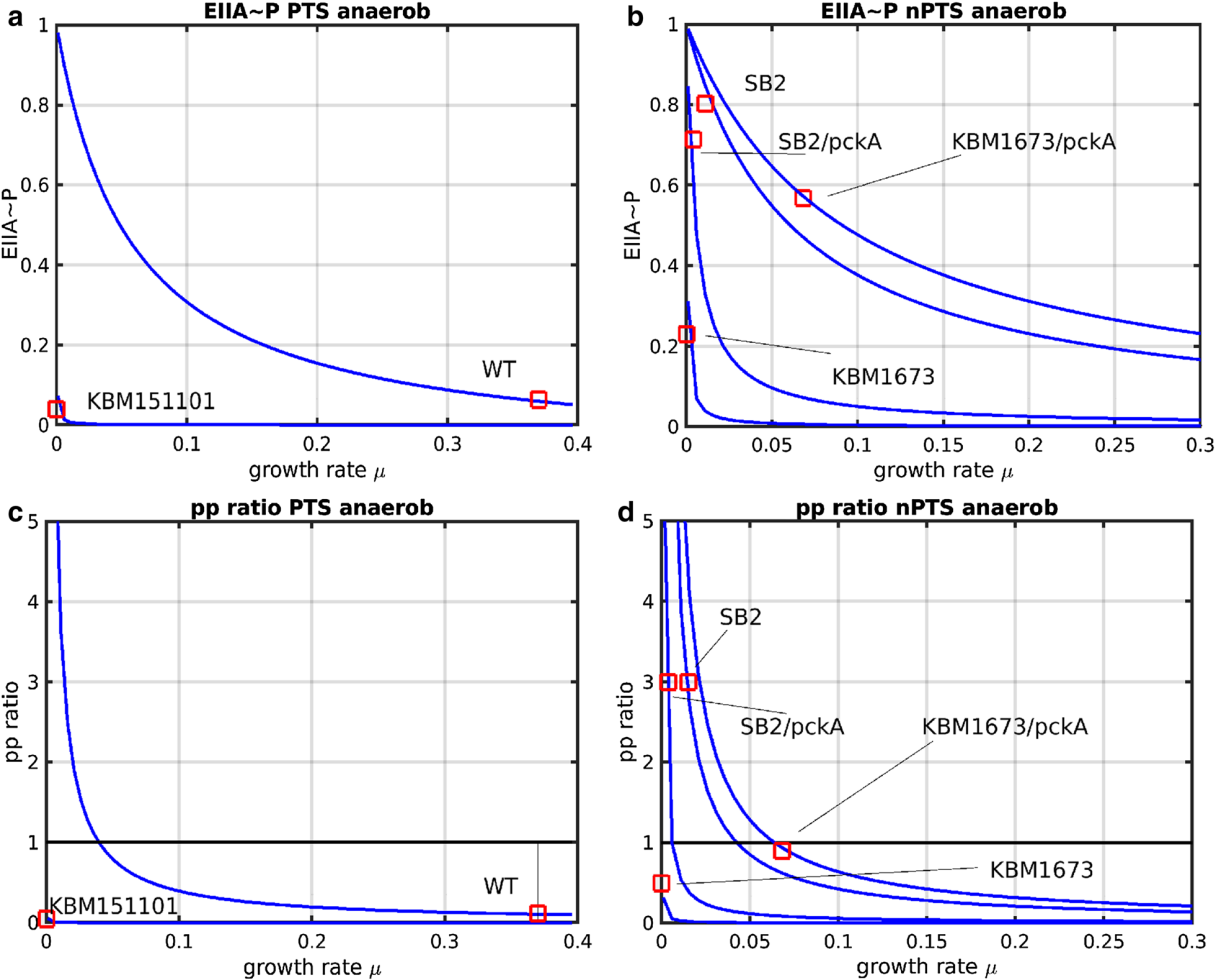
Course of the degree of phosphorylation of EIIA^Glc^ and PEP/pyruvate ratio over growth rate µ. Comparison of measurement and simulation. **a** Course of EIIA^Glc^ ~ P over growth rate for the anaerobic case and strains with PTS system. **b** Course of PEP/pyruvate ratio over growth rate for the anaerobic case and strains with PTS system. **c** Course of EIIA^Glc^ ~ P over growth rate for the anaerobic case and strains without PTS system. **d** Course of PEP/pyruvate ratio over growth rate for the anaerobic case and strains without PTS system. Green symbols are measured data points (see Tables 3, 5 in the main text)

To support the experimental findings, a simulation study was performed as described under Materials and Methods. For all strains, the experimentally determined glucose uptake rate and the degree of EIIA^Glc^ phosphorylation was provided to the model. The simulation results for the PEP/pyruvate ratio are shown in Fig. 6.

As one can see, a low PEP/pyruvate ratio (~ 1 or below 1) is predicted for strains showing pyruvate production (KBM151101, KBM1673) while a PEP/pyruvate ratio of about 3 is predicted for strains showing no pyruvate production (SB2 and SB2/pPck). It might hence be concluded that a low PEP/pyruvate ration of about 1 can be associated with pyruvate excretion.

Summarizing, the EIIA^Glc^ phosphorylation levels reflect current knowledge for the aerobic growth phase. The data for the anaerobic phase seem to indicate that in non-growing cells or under anaerobic conditions EIIA^Glc^ does not reflect glucose uptake but rather the potential accumulation of pyruvate inside the cell.

## Discussion

In order to improve succinate productivity by metabolic engineering of central pathways in *E. coli* different strategies have been explored [17, 38, 58, 59]. Several genetic manipulations are necessary to obtain a good production strain, because wild-type *E. coli* produce only minor amounts of succinate [48]. Typical manipulations are the deletion of pathways leading to alternative fermentation products such as lactate, ethanol, formate and acetate. As a consequence of this manipulations, the strains show low anaerobic growth rates or are not able to grow on glucose in minimal medium at all [38]. Although theoretically anaerobic growth is feasible, it is hardly observed without addition of complex medium components. Hence, rich media are used in most studies for improving growth and succinate production [7, 9]. Succinate production in *E*. *coli* has been studied in a number of different theoretical studies (for a review see [6]). However, for most of them an experimental validation is lacking. Typically, the different strategies result in strains carrying multiple mutations but the effect of a single mutation often was not investigated.

In this work we compared different strains for their succinate production using mineral salts medium. Strain KBM151101, unable to produce the fermentation products acetate, ethanol and formate, but still having a functional glucose-PTS, was not able to grow under anaerobic conditions. In fact, biomass was even dropping anaerobically. In two-stage cultivations of KBM151101, pyruvate accumulation was observed coupled to low ATP levels. This indicates that fluxes in this strain are not balanced. Most probably, the inability of producing enough ATP is limiting succinate production. This is also indicated by flux analysis. As KBM151101 takes up glucose by the Glc-PTS, glucose uptake is coupled to a conversion of PEP to pyruvate. In order to fulfill growth requirements and to regenerate NAD, pyruvate would have to be converted back to PEP but this is not possible, due to a lack of ATP. Similar results have been reported in previous studies dealing with succinate production in *E*. *coli*. These studies used strain NZN111, which has two mutations in lactate dehydrogenase and pyruvate formate lyase [7, 60]. Although mutations in NZN111 and KBM151101 are different, they have a similar effect, as both strain are unable to synthesize the main fermentation products.

We implemented a further mutation in KBM151101 by knocking out the Glc-PTS. The resulting strain, SB2, produced succinate from glucose anaerobically with a high yield of 1.24 mol/mol (about 80% of the maximal theoretical yield). This compares well to yields obtained from studies using similar strains carrying the *ptsI* or *ptsG* mutation as well as *pfl* mutations [40–42] In theory, inactivation of *ptsG* increases the PEP pool, because uptake of glucose is no longer coupled to the conversion of PEP to pyruvate. PEP hence becomes available for the formation of succinate. This observation has been described previously for *ptsG* mutants of NZM111 [17, 44]. Our hypothesis is that two main factors account for this observation. First, the PTS mutation decouples glucose uptake from PEP to pyruvate conversion. This enables fluxes from PEP to succinate without the need to input ATP for PEP synthase reaction. This is underlined by the results of flux analyses that predict improved succinate production as soon as part of the glucose is taken up by non-PTS systems. Secondly, Pck activity is needed to achieve high succinate yields. Flux analysis assigns an important function to PckA, too. This enzyme is able to couple PEP to oxaloacetate conversion with ATP synthesis. Our second hypothesis hence is that *pckA* is expressed in SB2. This hypothesis is supported by gene expression analyses, showing an increased *pckA* RNA level in SB2 compared to KBM151101 (Fig. 4). Pck is generally regarded as a gluconeogenic enzyme, which is not expressed in the presence of glucose [53, 54, 61]. Also *pck*A is known to be under control of CsrA [62]. It is hence tempting to speculate that deletion of *ptsG*, reducing glucose uptake, and the lack of ATP observed for KBM151101 under anaerobic conditions (Fig. 3) provoke expression of *pckA* in SB2, allowing for improved growth or survival, which is coupled to a high succinate yield. PckA overexpression in cells of SB2 decreased glucose uptake and succinate production rates, but increased succinate yield (Table 3). A positive effect of *pckA* overexpression was also reported by Kwon [43]. They observed this positive effect only in the presence of high carbonate concentrations, an observation that was verified by our experiments (data not shown).

In strains with *ptsG* deletion, the glucose facilitator protein, Glf, and the glucokinase, Glk, of *Z. mobilis* allow for improved glucose uptake [25, 63, 64]. Strain KBM1673, the Glf^+^ derivative of SB2, had a higher glucose utilization rate under aerobic condition than its parent strain, SB2. Contrary to expectation, glucose uptake rate as well as succinate yield and productivity of KBM1673 in the anaerobic phase decreased compared to SB2. This was due to significant excretion of pyruvate (Table 3). FBA (Fig. 5) shows that this strain is characterized by relatively high fluxes from PEP to pyruvate. We hypothesize that due to the presence of an efficient glucose uptake system that allows for a high growth rate in the aerobic preculture, an expression of *pckA* is either not possible or that the amount of Pck available is too low to direct the main flux towards succinate. In fact, we observed a lower expression of *pckA* for KBM1673 under anaerobic conditions than for SB2 (Fig. 4). Instead, the accumulating PEP is converted to pyruvate, a reaction that allows the cell to synthesize ATP, too. Pyruvate production might hence be considered an overflow reaction. A significant fraction of pyruvate is found in the outside medium (Table 3, Fig. 5). If the flux through Pck is enhanced by overexpression of the enzyme, high succinate productivity is achieved. An alternative explanation for the increased pyruvate production might be the involvement of an alternative PTS in intracellular glucose phosphorylation. In this case the respective PTS system would not be able to transport extracellular glucose but to phosphorylate glucose that accumulates intracellularly due to the presence and activity of Glf.

Figure 5 summarizes our theoretical and experimental findings. The bars represent the ratio of the fluxes at node PEP and shows that strains with a high yield (red curve) direct the flux mainly from PEP to oxaloacetate while a smaller part is going to pyruvate. Strain KBM1673 with PckA shows the highest productivity, however, as can be seen in Fig. 5, a large fraction from the flux to pyruvate is excreted, which can be interpreted as metabolic overflow. This points to a bottleneck in the drain from pyruvate to acetyl-CoA (the non-hatched part in the yellow bars is nearly equal for all strains except KBM151101).

In summary, this study demonstrates that it is possible to achieve a good succinate yield with minimal number of interventions also for cultivation in minimal media. To achieve high succinate yields, it is important to direct a significant flux through Pck, in order to gain ATP and in order to avoid disadvantageous pyruvate production. A high succinate yield was achieved by an engineered strain SB2 with Pck overexpression by using two-stage cultivations. This strain reaches a succinate yield of 1.4 mol/mol glucose, which is near to the theoretical maximum that could be achieved (theoretical maximum 1.7 mol/mol glucose). A lower yield but a much better productivity was achieved in KBM1673 with *pckA* overexpression. Based on our results a further strain improvement would require to fine-tune incoming glycolytic fluxes with the fluxes at the PEP/pyruvate node, especially with fluxes through Pck to avoid pyruvate accumulation and to allow for ATP and succinate synthesis.

## Additional files

**Additional file 1: Table S1.** Primers used in this study. **Table S2.** Single measurements of EIIA^Glc^ phosphorylation state during two-stage cultivations. **Figure S1.** Pictures of chemiluminescence detection of Western Blots. **Figure S2.** Results from ANOVA analysis of intracellular ATP levels.

**Additional file 2.** Model structure and FBA results  for the different strains.
