# Supplementary material for: Type and capacity of glucose transport influences succinate yield in two-stage cultivations
Source: Microb Cell Fact. 2018 Aug 28;17:132. doi: 10.1186/s12934-018-0980-1 (PMC6112142; doi:10.1186/s12934-018-0980-1)
Supplement: Supplementary file 1 — Additional file 1: Table S1. Primers used in this study. Table S2. Single measurements of EIIAGlc phosphorylation state during two-stage cultivations. Figure S1. Pictures of chemiluminescence detection of Western Blots. Figure S2. Results from ANOVA analysis of intracellular ATP levels. [file 12934_2018_980_MOESM1_ESM.docx]

**Table S1: Primers used in this study**

| \|  \|  \|  \| \| --- \| --- \| --- \| \| **Name** \| **Sequence (5´...3´)** \| **Application** \| \| ackA-for-ko \| TTTTTTAGCCACGTATCAATTATAGGTACTTCCatggtgtaggctggagctgcttcg \| *ackA* deletion \| \| ackA-rev-ko \| GCTGGCGGTGTGAAATCAGGCAGTCAGGCGGCTCGCcatatgaatatcctcctta \| *ackA* deletion \| \| pta-for-ko \| CGCCAAATCGGCGGTAACGAAAGAGGATAAACCGTGgtgtaggctggagctgcttcg \| *pta* deletion \| \| pta-rev-ko \| CGGATGATGACGAGATTACTGCTGCTGTGCAGACTGcatatgaatatcctcctta \| *pta* deletion \| \| adhE-for-ko \| GAGCAGATGATTTACTAAAAAAGTTTAACATTATCAGGAGAGCATTATGgtgtaggctggagctgcttcg \| *adhE* deletion \| \| adhE-rev-ko \| CCGTTTATGTTGCCAGACAGCGCTACTGAttaAGCGGATTTTTTCGCTTTcatatgaatatcctcctta \| *adhE* deletion \| \| ldhA_for-ko \| GCTTAAATGTGATTCAACATCACTGGAGAAAGTCTTGTGTAGGCTGGAGCTGCTTCG \| *ldhA* deletion \| \| ldhA-rev-ko \| GAATCAGCTCCCCTGGAATCAGGGGAGCGGCAAGACATATGATATCCTCCTTA \| *ldhA* deletion \| \| delta-lacA-rev \| ggcaatttttataatttaaactgacgattcaactttTGTAGGCTGGAGCTGCTTCG \| *lacA-I* deletion \| \| lacI-k.o \| agcgcccggaagagagtcaattcagggtggtgaatgcatatgaatatcctcctta \| *lacA-I* deletion \| \| ptsG-for-ko \| AAAAAGCACCCATACTCAGGAGCACTCTCAATTatggtgtaggctggagctgcttcg \| *ptsG* deletion \| \| ptsG-rev-ko \| GCCTTAGTCTCCCCAACGTCTTACGGAttaGTGGTTattccggggatccgtcgacc \| *ptsG* deletion \| \| pAH162_ rev \| ACCTGCAGGCATGCAAGC \| pAH162-glf-glk \| \| PsrcK_fwd \| tgcatgcctgcaggtTGGCCTTCTTCGCTCAGTAAC \| pAH162-glf-glk \| \| PsrcK_rev \| aatcgccgGGATCCGCTCCTGTTGCA \| pAH162-glf-glk \| \| glf-glk_fwd \| gcggatccCGGCGATTGTAAGATTTAC \| pAH162-glf-glk \| \| glf-glk_rev \| tgtcaaacatgagaattcCCTCTTAAATTCAGTTCATAATATTAAAAAATATTATTC \| pAH162-glf-glk \| \| pAH162_ fwd \| GAATTCTCATGTTTGACAGCTTATCACTG \| pAH162-glf-glk \| \| delta-glk_zm_ for \| TAAGAGGGAATTCTCATGTT \| *glk* deletion from pAH162-glf-glk \| \| delta-glk-Zm-rev \| TTCTAAAATCAGAAGGCTCAA \| *glk* deletion from pAH162-glf-glk \| \| pck-for-NdeI \| aatcatatgAGGAGAaattatgCGCGTTAACAATGGTTTG \| *pckA* amplification \| \| pck-rev-PstI \| aatctgcagAATCAttaCAGTTTCGGACC \| *pckA* amplification \| \| rpoD-f-Real \| CGCGCACCATCCGTATTC \| RealTme RT PCR \| \| rpoD-r-Real \| TTGGCGATCTTCAGCACTTTG \| RealTme RT PCR \| \| ihfB-f-real \| gccaagacggttgaagatgc \| RealTme RT PCR \| \| ihfB-r-real \| gagaaactgccgaaaccgc \| RealTme RT PCR \| \| ybhc-for-real \| GTCGCGGCGCAGTGGTGTT \| RealTme RT PCR \| \| ybhC-rev-real \| ACGGCTGTTTACGGCGAGGAA \| RealTme RT PCR \| \| pckA-f-real \| ACATGTTTATTCGCCCGAGC \| RealTme RT PCR \| \| pckA-r-real \| CTGTTCTTTCCACTGCGGGT \| RealTme RT PCR \| \| ppc-f-real \| CCGCGATTGGCCATTCTTCTC \| RealTme RT PCR \| \| ppc-r-real \| TCGCCAGCACCACTTTGATGTC \| RealTme RT PCR \| |
| --- | --- | --- | --- | --- | --- | --- | --- | --- | --- | --- | --- | --- | --- | --- | --- | --- | --- | --- | --- | --- | --- | --- | --- | --- | --- | --- | --- | --- | --- | --- | --- | --- | --- | --- | --- | --- | --- | --- | --- | --- | --- | --- | --- | --- | --- | --- | --- | --- | --- | --- | --- | --- | --- | --- | --- | --- | --- | --- | --- | --- | --- | --- | --- | --- | --- | --- | --- | --- | --- | --- | --- | --- | --- | --- | --- | --- | --- | --- | --- | --- | --- | --- | --- | --- | --- | --- | --- | --- | --- | --- | --- | --- | --- | --- | --- | --- | --- | --- | --- | --- | --- | --- |

**Table S2: Single measurements of EIIA^Glc^ phosphorylation state during two-stage cultivations**

|  | **MG1655** | | **KBM**  **151101** | | **SB2** | | | **KBM1673** | | | **SB2/pPck** | | | **KBM1673/pPck** | | |  |
| --- | --- | --- | --- | --- | --- | --- | --- | --- | --- | --- | --- | --- | --- | --- | --- | --- | --- |
|  | aer | anae | aer | anae | | aer | anae | | aer | anae | | aer | anae | | aer | anae | |
|  | 3.2 | 6.4 | 1.7 | 5.3 | | 92.2 | 78.1 | | 63.2 | 27.6 | | 88.1 | 73.9 | | 71.4 | 67.7 | |
|  | 5.1 | 3.4 | 3.1 | 6.3 | | 90.4 | 82.5 | | 80.9 | 19.1 | | 87.4 | 69.0 | | 47.3 | 46.0 | |
|  |  |  | 1.6 | 1.9 | |  |  | |  |  | | 83.0 |  | | 55.0 |  | |
|  |  |  |  |  | |  |  | |  |  | | 75.0 |  | | 72.0 |  | |
| **Av** | **4.2** | **4.9** | **2.1** | **4.5** | | **91.3** | **80,3** | | **72.1** | **23,3** | | **83.4** | **71.5** | | **61.4** | **56.9** | |
| **StDev** | 1.3 | 2.1 | 0.8 | 2.3 | | 1.3 | 3,1 | | 12.5 | 6,0 | | 6.0 | 3.5 | | 12.3 | 15.3 | |

Shown are the values for the phosphorylated part of EIIA^Glc^ determined from single growth curves. From each curve two to three samples were analyzed. The data shown in the table above represent average values of those repeats from one curve. aer: depicts the aerobic growth phase, anae: denotes the second, anaerobic stage.

**
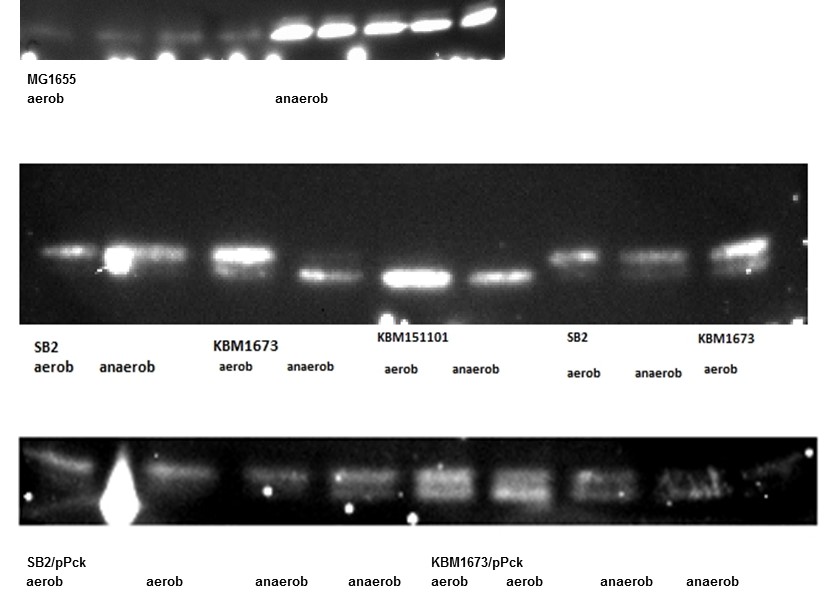
**

**Figure S1: Pictures of chemiluminescence detection of Western Blots.** Extracts of the different strains indicated in the figure were subjected to gel electrophoresis and Western Blotting with anti-EIIA^Glc^ serum. EIIA^Glc^ runs in two forms, an upper band corresponding to phosphorylated EIIA^Glc^ and a lower band corresponding to dephosphorylated EIIA^Glc^. Both bands of a lane were quantified against each other. For details see Materials and Methods.

**
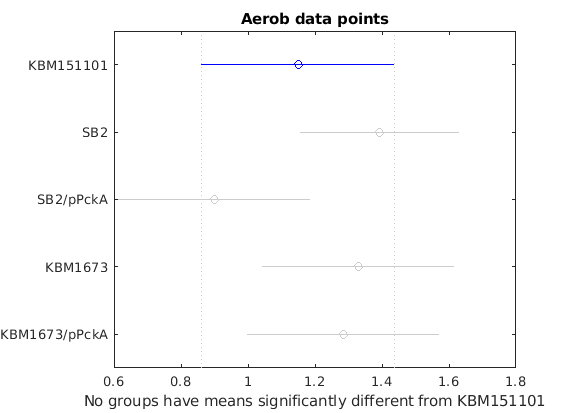
**

**
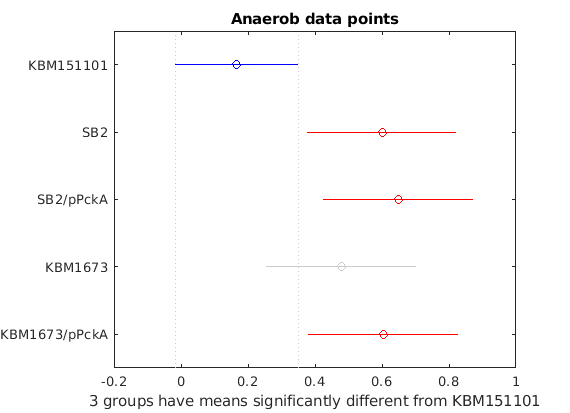
**

**Figure S2: Results from ANOVA analysis of intracellular ATP levels.** Shown are the data from the aerobic (uupper picture) and the anaerobic (lower picture) stage. While no significant differences in the ATP concentration can be observed for the aerobic stage, under anaerobic conditions ATP levels of KBM151101 are significantly lower then those of the other strains.

A pairwise comparison was performed by the calculation of the difference of the mean of the two date points and the corresponding 99% confidence interval. If the value “0” is not in the interval, the two mean values are sigifically different**.**
